# Primary Central Nervous System Lymphoma  with Peripheral Nerve Involvement:  Case Report

**DOI:** 10.7759/cureus.5675

**Published:** 2019-09-16

**Authors:** Yoshimasa Mori, Koh Yamamoto, Ako Ohno, Masaharu Fukunaga, Atsushi Nishikawa

**Affiliations:** 1 Radiation Oncology and Neurosurgery, Center for Advanced Image-guided Radiation Therapy, Shin-yurigaoka General Hospital, Kawasaki, JPN; 2 Neurosurgery, Shin-yurigaoka General Hospital, Kawasaki, JPN; 3 Internal Medicine, Department of Hematology, Shin-yurigaoka General Hospital, Kawasaki, JPN; 4 Pathology, Division of Pathological Examination, Shin-yurigaoka General Hospital, Kawasaki, JPN; 5 Radiation Oncology, Center for Advanced Image-guided Radiation Therapy, Shin-yurigaoka General Hospital, Kawasaki, JPN

**Keywords:** lymphoma, neurolymphomatosis, chemotherapy, radiation treatment, diffuse large b-cell lymphoma (dlbcl), peripheral nerve tumors, craniospinal irradiation, fdg-pet, central nervous system (cns) tumor, central nervous system lymphoma

## Abstract

A 50-year-old man presented with dizziness and hearing disturbance in the right ear. Magnetic resonance imaging (MRI) revealed a well-enhanced mass lesion in the right cerebellopontine (CP) angle that appeared to originate in the cerebellum. A surgical specimen obtained at the subtotal resection with craniotomy revealed a diffuse large B-cell lymphoma (DLBCL). During the three courses of chemotherapy with high-dose methotrexate (MTX) with leucovorin rescue, he developed a right abducens palsy, left oculomotor palsy, left facial palsy, right trigeminal sensory disturbance, and paraparesis. Although the brain MRI showed that the CP angle tumor had disappeared completely following chemotherapy, enhanced lesions along the cauda equina were detected on a lumbar spine MRI. FDG-PET (18 F-fluorodeoxyglucose positron emission tomography) revealed multiple high-uptake abnormalities in the cranial nerves and spinal nerves. Tumor cells were found in the cerebrospinal fluid specimen from a lumbar puncture. Craniospinal irradiation was performed, including all the abnormal FDG high-uptake areas, and was effective in relieving the patient’s symptoms. On FDG-PET, the high-uptake abnormalities in the peripheral nerves disappeared. However, five weeks after the irradiation, he developed right trigeminal sensory disturbance, hoarseness, dysphagia, and right arm pain. FDG-PET disclosed multiple high-uptake abnormalities in more peripheral portions of the cranial nerves and spinal nerves. Chemotherapy with rituximab, cyclophosphamide, doxorubicin hydrochloride, vincristine (Oncovin®), and prednisolone (R-CHOP) was then resorted to which mitigated his symptoms. On follow-up FDG-PET, the high-uptake abnormalities in the peripheral nerves disappeared again.

## Introduction

Primary non-Hodgkin’s lymphoma (NHL) of the central nervous system (CNS) is uncommon and generally affects the brain [1]. Diffuse large B-cell lymphoma (DLBCL) is the most common form of CNS NHL. Only 1% of primary CNS NHL emerges in the spinal cord. A syndrome of lymphoma and leukemic infiltration of the cranial or peripheral nerves is called neurolymphomatosis (NL) [2-3]. It is a rare clinical entity, with an incidence of 0.2% in all NHL patients [3]. Several studies have reported primary and secondary NL with single and multiple cranial or spinal nerve involvement [1-9]. We present a case of CNS NHL developing NL subsequently during the initial courses of chemotherapy following subtotal surgical resection of the brain lymphoma. As far as we know, this is the first reported case in which primary brain NHL with NL, without other visceral lesions, manifested subsequently during the initial chemotherapy.

## Case presentation

**Figure 1:**
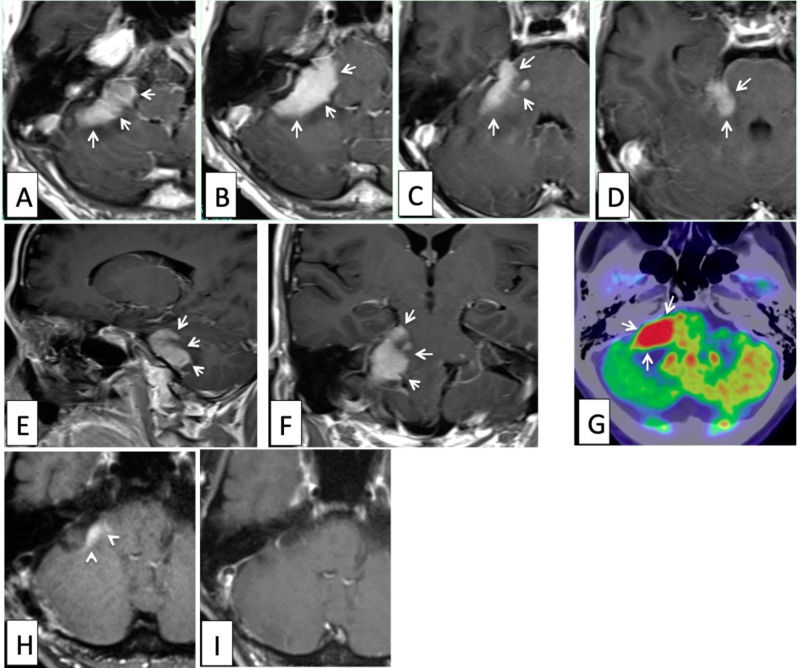
Magnetic resonance imaging (MRI) demonstrating a well-enhanced mass lesion in the right cerebellopontine (CP) angle appearing to have originated in the cerebellum Axial (A, B, C, D), sagittal (E), and coronal (F) images of gadolinium (Gd)-enhanced T1-weighted MRIs at presentation. A well-enhanced tumor was located mainly in the right cerebellopontine angle brain parenchyma (white arrows). It showed high-uptake of fluorodeoxyglucose (FDG) on positron emission tomography (PET) (G, white arrows). Only a small part of the tumor remained on a postoperative axial image of the Gd-enhanced MRI (H, white arrowheads). No enhanced mass was seen on an axial image of the MRI (I) after chemotherapy using high-dose methotrexate (MTX).

A 50-year-old man suffered from dizziness and hearing disturbance in the right ear. Magnetic resonance imaging (MRI) demonstrated a well-enhanced mass lesion in the right cerebellopontine (CP) angle, which appeared to have originated in the cerebellum (Figure 1). 

**Figure 2:**
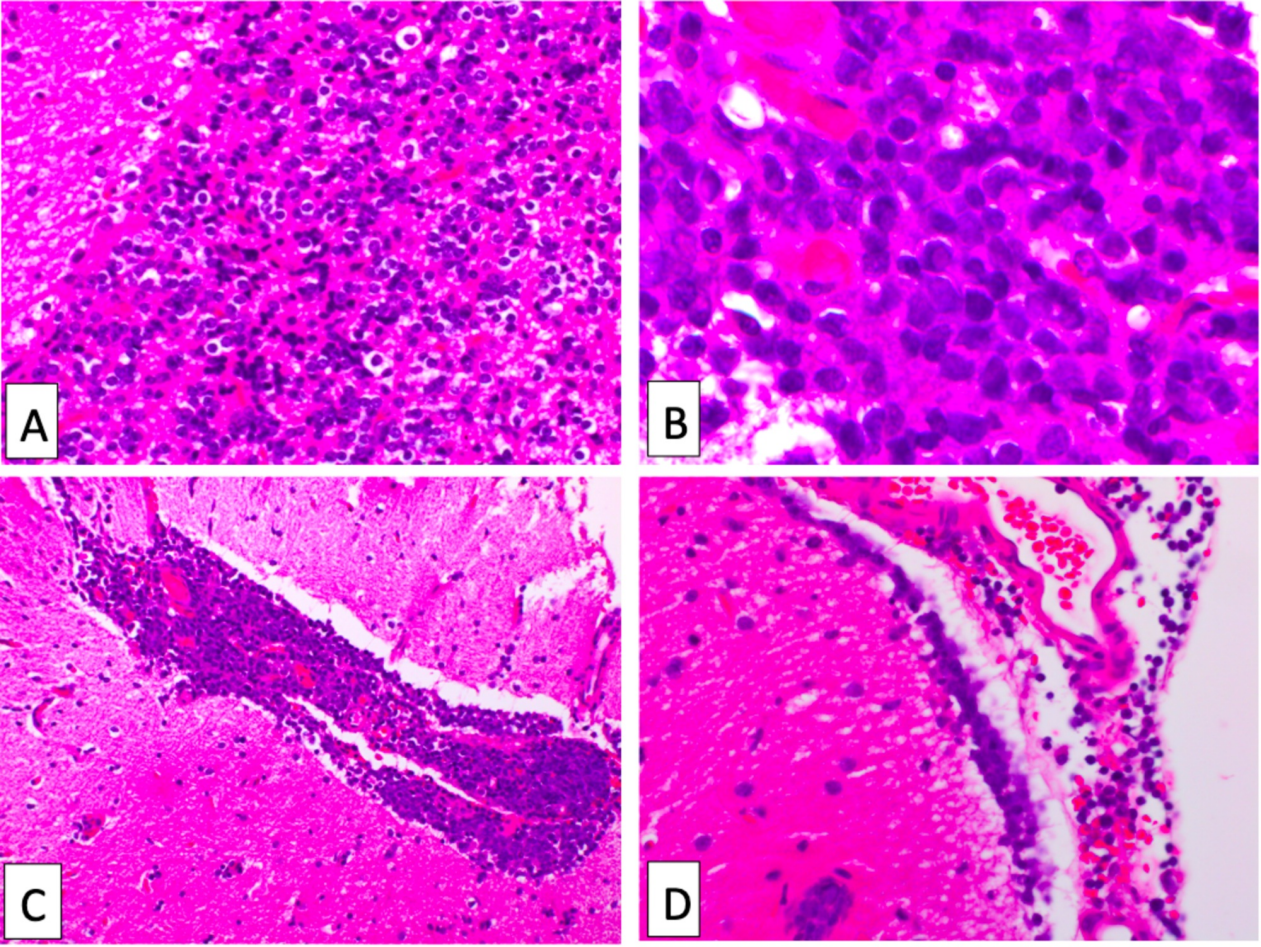
Hematoxylin and eosin (H&E) stains of atypical lymphoid cells A: Diffuse proliferation of atypical lymphoid cells; B: Atypical lymphoid cells have medium to large nuclei, prominent nucleoli, and scant cytoplasm; C and D: Perivascular proliferation of atypical lymphoid cells in the subarachnoid space (C) and subpial space (D).

**Figure 3:**
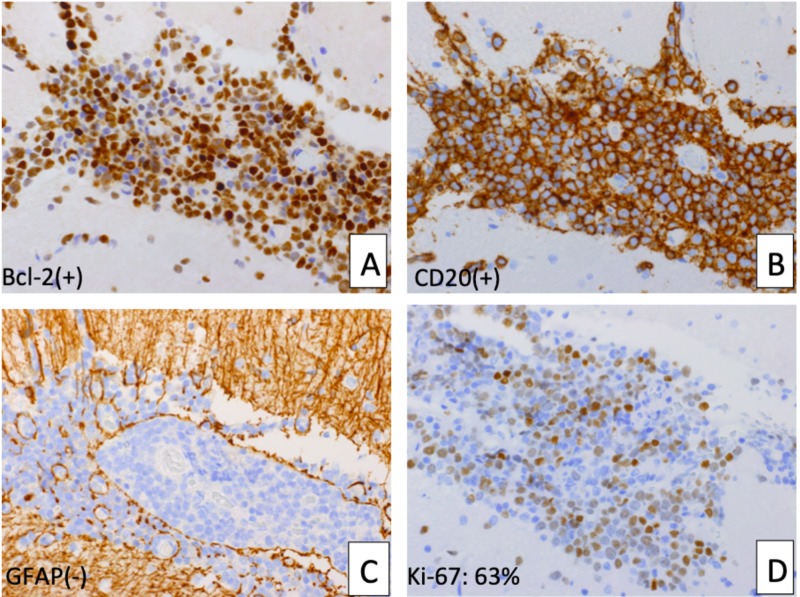
Immunostaining of the atypical lymphoid cells A and B: Atypical lymphoid cells are diffusely positive for Bcl-2 (A) and CD20 (B); C: Atypical lymphoid cells are negative for glial fibrillary acidic protein (GFAP); D: Ki-67 positive index is 63%. Bcl-2: B-cell chronic lymphocytic leukemia/lymphoma 2; CD20: cluster of differentiation 20; Ki-67: one of the proliferative proteins

The surgical specimen taken at the time of the subtotal resection with craniotomy revealed a DLBCL (Figures 2-3).

**Figure 4:**
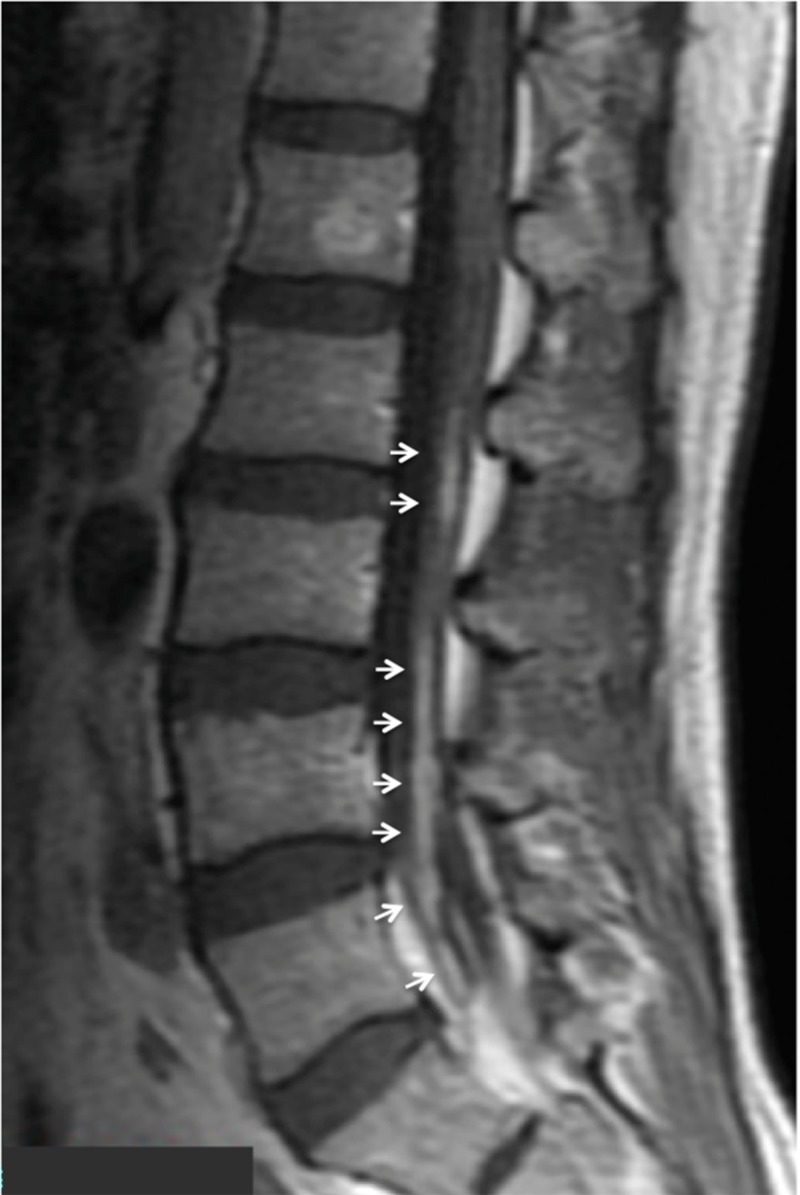
A sagittal view of lumbar magnetic resonance imaging (MRI) that disclosed gadolinium-enhancement in the cauda equina (white arrows).

During the three courses of chemotherapy with high-dose methotrexate (MTX) with leucovorin rescue, he developed a right abducens palsy, left oculomotor palsy, left facial palsy, right trigeminal sensory disturbance, and paraparesis. Although the CP angle tumor on the brain MRI showed complete regression following chemotherapy (Figure 1), enhanced lesions along the cauda equina were detected on lumbar spinal MRI (Figure 4).

**Figure 5:**
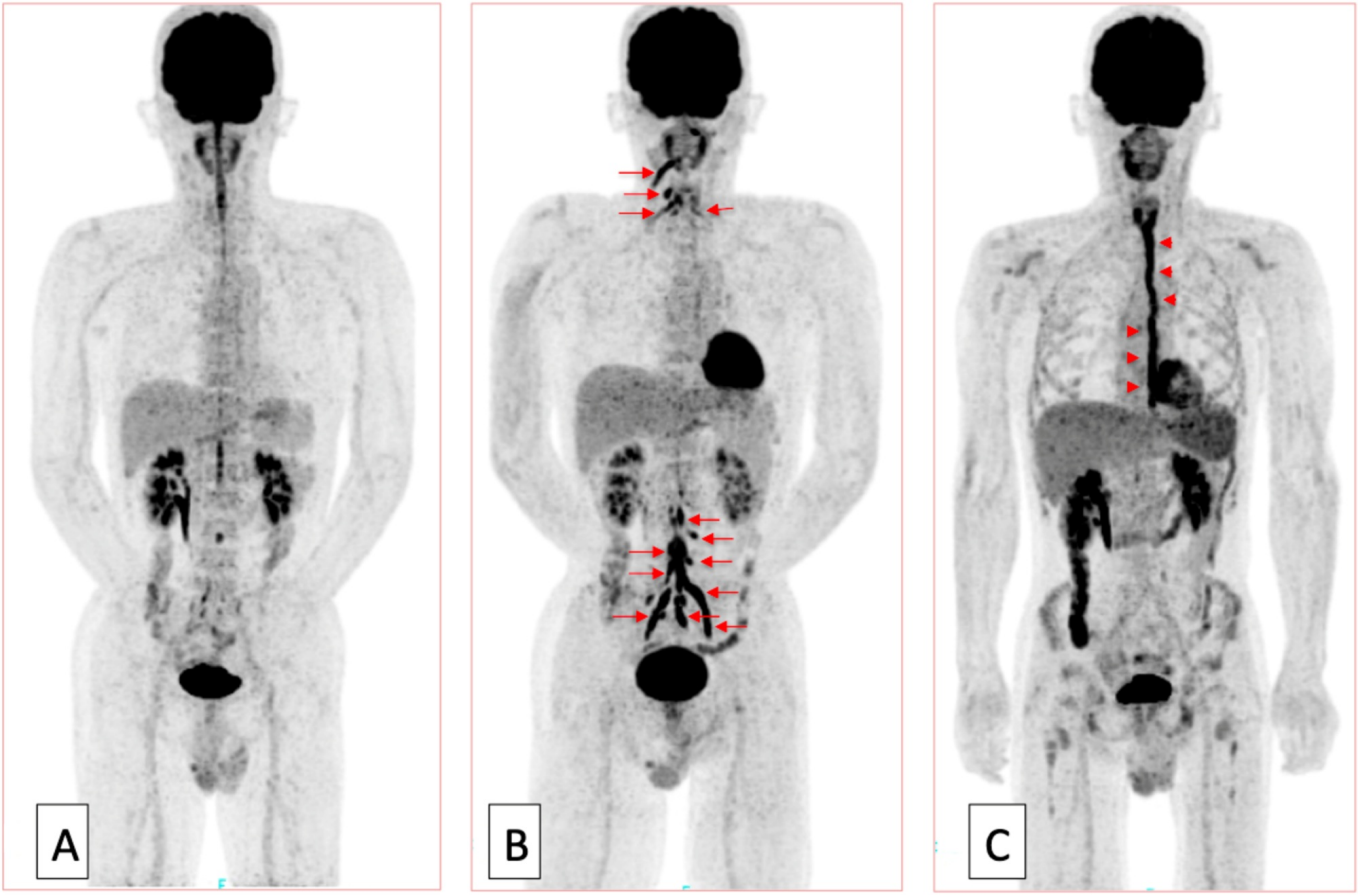
Fluorodeoxyglucose positron emission tomography (FDG-PET) at presentation, after chemotherapy, and after craniospinal irradiation FDG-PET at presentation (A), following chemotherapy with high-dose methotrexate (MTX) (B), and after craniospinal irradiation (C). FDG-PET after high-dose MTX showed accumulation of FDG in the nerve roots somewhat extending somewhat further into the peripheral portions (B, orange arrows), which had not been noted before previously (A). In addition, this high-uptake area almost completely disappeared after conventional radiation therapy (C).  Accumulation of FDG along the esophagus was due to radiation esophagitis (C, orange arrowheads).

**Figure 6:**
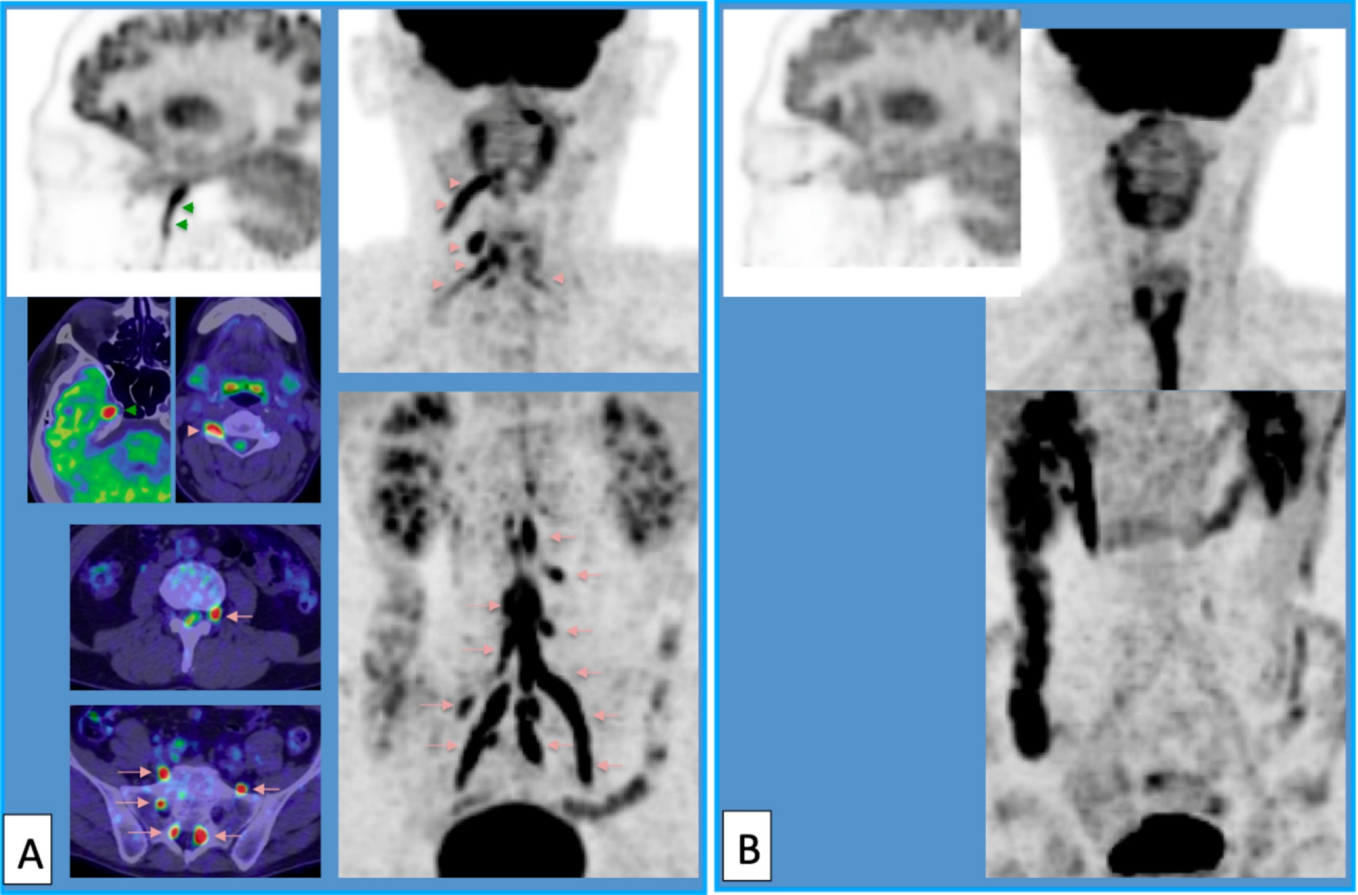
High magnification of fluorodeoxyglucose positron emission tomography (FDG-PET) after chemotherapy and after craniospinal irradiation High magnification of FDG-PET after chemotherapy with high-dose methotrexate (MTX) (A) and after irradiation (B). Accumulation of FDG developed in the right trigeminal nerve (green arrowheads) and cervical (red arrowheads) and lumbosacral (red arrows) roots and plexus (A). These high-uptake areas disappeared after irradiation (B).

Fluorodeoxyglucose positron emission tomography (FDG-PET) revealed multiple high-uptake abnormalities in the cranial nerves and spinal nerves (Figures 5-6).

**Figure 7:**
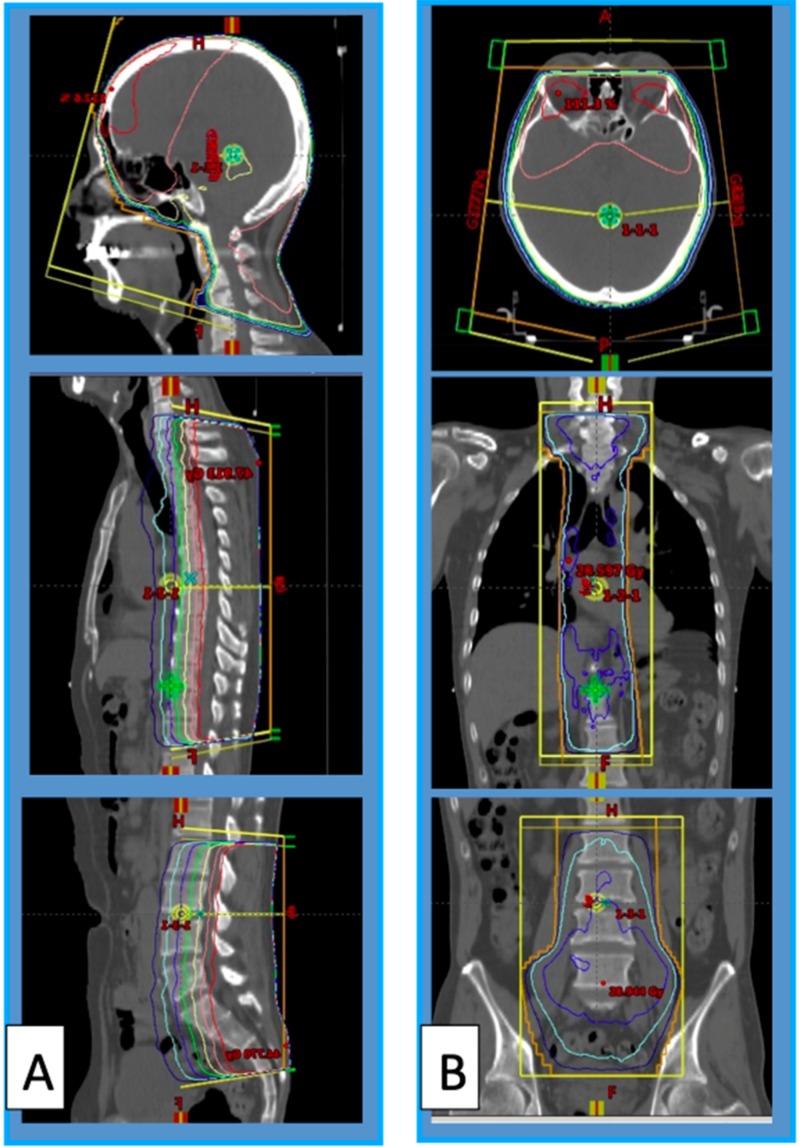
Dose planning of craniospinal irradiation Lateral views (A), axial (B, upper), and frontal views (B, middle and lower). High-uptake areas of fluorodeoxyglucose (FDG) were covered by 95% dose line.

**Figure 8:**
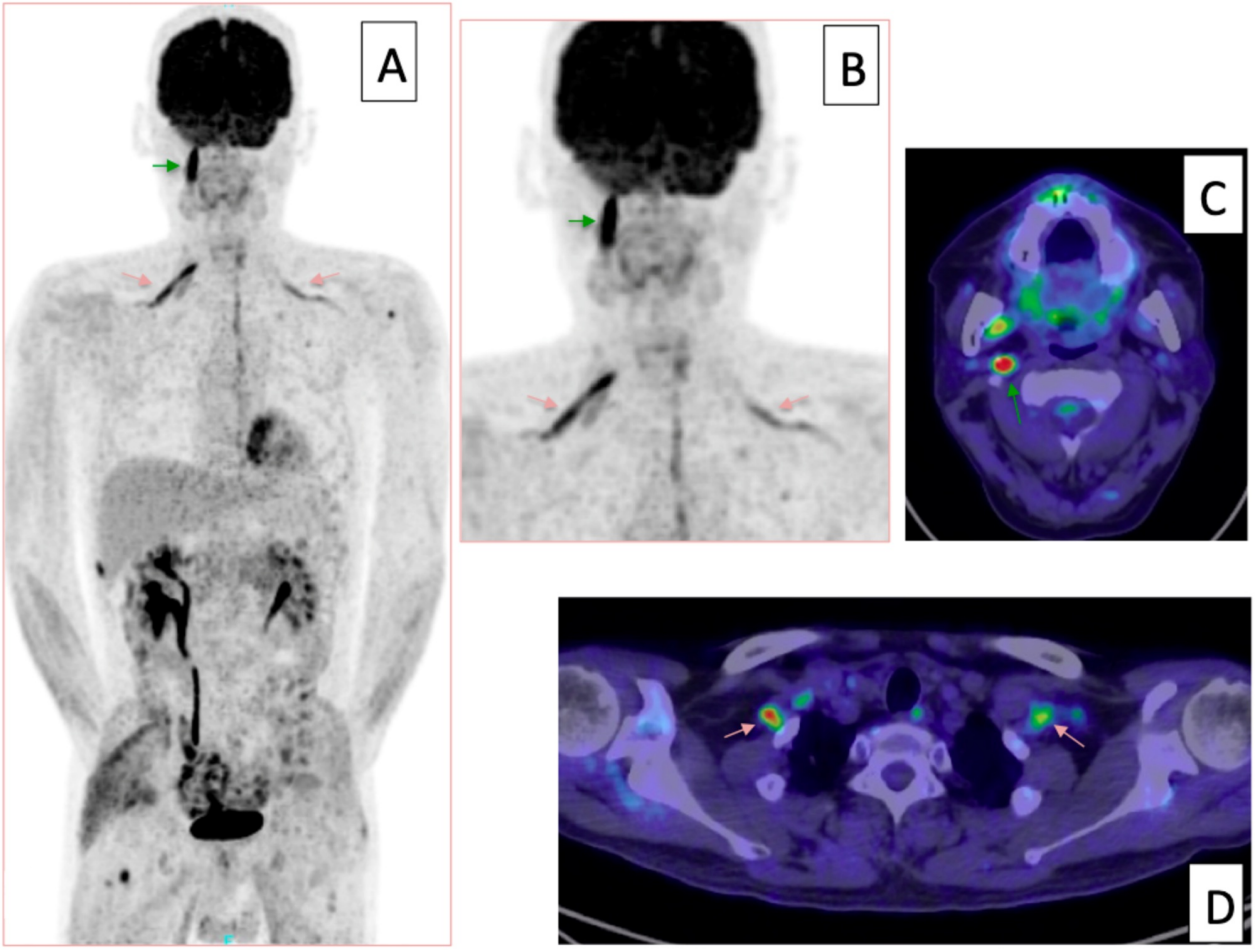
Subsequent fluorodeoxyglucose-positron emission tomography (FDG-PET) five weeks after craniospinal irradiation Fluorodeoxyglucose (FDG) accumulated in the right lower cranial nerve root (A, B, C, green arrows) and brachial plexus (A, B, D, red arrows). The radiation-induced esophagitis was almost totally relieved.

Tumor cells were found in the cerebrospinal fluid specimen from the lumbar puncture. Craniospinal irradiation was performed, including all the abnormal FDG high-uptake areas (Figure 7), and effectively relieved the patient’s symptoms. On the FDG-PET scan, the high-uptake abnormalities in the peripheral nerves disappeared. However, five weeks after the irradiation, he developed a right trigeminal sensory disturbance, hoarseness, dysphagia, and right arm pain. FDG-PET disclosed multiple high-uptake abnormalities in more peripheral portions of the cranial and spinal nerves (Figure 8).

Chemotherapy consisting of R-CHOP (rituximab, cyclophosphamide, doxorubicin hydrochloride, vincristine (oncovin), and prednisolone) was then administered, which began two weeks after the development of recurrent symptoms. We chose R-CHOP, other than ordinal salvage regimens for CNS lymphoma such as high-dose cytosine arabinoside, because his general condition was not good enough after esophagitis was relieved. His symptoms again improved in two weeks after the beginning of R-CHOP. On the FDG-PET scan, taken three months after the beginning of R-CHOP (after three courses of R-CHOP), the high-uptake abnormalities in the peripheral nerves again disappeared. At that time his neurological conditions were good only with dysesthesia in the lower extremities.

## Discussion

The lymphomatous infiltration of a nerve root (the cranial or peripheral nerve or multiple nerves) is known as NL or neurolymphomatosis [2]. It is a rare neurologic complication of lymphoma that is poorly recognized by clinicians [2].

Haydaroglu Sahin et al. [4] and Katyal et al. [5] reported a case of NL in multiple cranial nerves. Bourque et al. reported a case of NL in the brachial plexus [6]. Khandelwal et al. presented a case of NL in the left C4 and C5-C8, and L4-L5 and S1-S2 [2]. Broen et al. [1], Sasaki et al. [3], and Marquardt et al. [7] reported cases of NL in the cauda equina or lumbosacral root/plexus. Misdraji et al. reported four cases of NL in the sciatic nerve (two cases), radial nerve, or sympathetic chain and spinal nerve [8]. Del Grande et al. reported a case of NL in the femoral nerve [9]. 

Gd-enhanced MRI and FDG-PET are the imaging modalities of choice for evaluation of patients with lymphoma and suspected neural involvement [2]. NL is diagnosed by the demonstration of enhancement of nerve roots on MRI of the brachial, lumbosacral plexus, peripheral nerves, or by increased hypermetabolic activity along the course of affected nerves on FDG-PET [2]. Treatment of NL consists of focal radiotherapy and chemotherapy [2]. In our case, NL developed and worsened during chemotherapy with high-dose MTX. Craniospinal irradiation, followed by R-CHOP, were then effective. 

## Conclusions

A case of primary central nervous system lymphoma presenting with peripheral nerve involvement was described. Recognizing these conditions with an understanding of the rare clinical entity of neurolymphomatosis is necessary for early diagnosis of similar cases. Detailed neurological examination and imaging, including FDG-PET, would be essential to grasp the extent of the disease. Timely assessment with chemotherapy and irradiation, if possible, might improve the patient's neurological status.
